# Solvothermal synthesis of uniform bismuth nanospheres using poly(*N*-vinyl-2-pyrrolidone) as a reducing agent

**DOI:** 10.1186/1556-276X-6-66

**Published:** 2011-01-12

**Authors:** Jiliang Wu, Fan Qin, Zhong Lu, Hai-Jian Yang, Rong Chen

**Affiliations:** 1 Key Laboratory for Green Chemical Process of Ministry of Education and School of Chemical Engineering & Pharmacy, Wuhan Institute of Technology, Wuhan, 430073, P. R. China; 2 Key Laboratory of Catalysis and Materials Science of the State Ethnic Affairs Commission & Ministry of Education, College of Chemistry and Materials Science, South-Central University for Nationalities, Wuhan, 430074, P. R. China

## Abstract

Uniform bismuth nanospheres were successfully prepared from bismuth nitrate in the presence of poly(*N*-vinyl-2-pyrrolidone) (PVP) by solvothermal process. The product was characterized by powder X-ray diffraction, scanning electron microscopy, transmission electron microscopy, selected area electron diffraction, and energy-dispersive X-ray. PVP plays a critical role both as a reducing agent and a capping agent in the formation of bismuth nanospheres. Shape and size of bismuth nanospheres could be tuned by changing the employed PVP/bismuth salt ratio. It was also found the solvent had an effect on the morphologies of bismuth nanomaterials. The possible formation and growth mechanism of bismuth nanospheres were also discussed and proposed to explain the reduction step.

## Introduction

Recently, metal nanostructures with functional properties have been extensively studied due to their wide applications. Among the various metal nanostructures, monodisperse metallic nanospheres have attracted considerable attention because of their unique optical, catalytic, as well as novel chemical and biological properties [1-8]. For example, the controlled silver nanospheres were applied for optical studies and antibacterial applications [9,10]. Thus, much effort has been devoted to the preparation of new metal nanospheres and their functional transformation [11-14]. As a semimetal, bismuth has unusual properties due to its highly anisotropic Fermi surface, low carrier densities, small carrier effective masses, and long carrier mean free path [15]. Bismuth nanostructures have received steadily growing interests, since they play important roles in many diverse applications [16,17]. Different bismuth nanomaterials, such as single-crystal bismuth thin film and nanowire, have been investigated for their large magnetoresistance, finite-size effects, and enhanced thermal conductivity [18-20]. It was also reported that spherical bismuth nanomaterials were a good catalyst for the growth of SnS_2 _nanotubes and germanium nanowires [21,22]. Up to now, a wide variety of bismuth nanostructures such as nanoparticles [23], nanowires [24], nanorods [25], nanotubes [26-28], triangular nanoplates [29], bunch-like nanostructures [17], nanocubes [30], as well as nanospheres (>100 nm) [31-34] have been prepared by using different methods. Among these methods, solvothermal synthesis has become an important and promising approach to prepare controlled inorganic nanocrystals [35-37].

In the reported literatures, the alcohol [normally ethylene glycol (EG)] was used as both solvent and reducing agent to generate bismuth nanospheres with a wide size distribution [31-34]. Wang and Xia [38] have developed the synthesis of uniform bismuth nanospheres by thermal decomposition bismuth acetate in EG under the protection of nitrogen. The use of protection of inactive gas and organic metal precursor, however, made it complicated and costly. Hence, a simple, low-cost approach for the preparation of uniform bismuth nanospheres is highly desired.

In this communication, we describe a solvothermal process for the synthesis of uniform bismuth nanospheres in the presence of poly(*N*-vinyl-2-pyrrolidone) (PVP). In most cases, PVP was used as a protecting agent to cap and stabilize the particles to avoid aggregation in the solution [39]. It was also reported that PVP was used as reducing agent in the preparation of noble metallic nanostructures [40,41]. In this synthesis, PVP acts as both reducing and capping agent. To the best of our knowledge, there are few publications relevant to the synthesis of bismuth nanomaterials using PVP as a reducing agent.

## Experimental

### Chemicals

Bismuth nitrate pentahydrate (Bi(NO_3_)_3_·5H_2_O) was purchased from Fuchen Chemicals Reagent (China). *N*,*N*-dimethylformamide (DMF), ammonia, mannitol, and EG were purchased from Sinopharm Chemical Reagent (China); PVP (with average *M*_w _of 10,000) was purchased from Sigma-Aldrich Inc. All the reagents were analytical grade and used directly without further purification.

### Synthesis of uniform bismuth nanospheres

In a typical procedure, 0.5 mmol Bi(NO_3_)_3_·5H_2_O was dissolved in 12.5 mL of 0.1 mol/L mannitol solution, then 7.5 mL NH_3_·H_2_O (5 mol/L) was added to the solution slowly with vigorously stirring. After stirring for about 15 min, the white precipitation was collected and washed with deionized water by centrifugation for five times. Then the obtained white precipitation and 0.155 g of PVP were introduced into 35 mL EG. The mixture was stirred and sonicated until all the chemicals were well dispersed. Then it was transferred into a stainless steel autoclave with Teflon liner. The autoclave was sealed and maintained at 180°C for 12 h. After cooling to room temperature, the obtained black solution was centrifuged and the solid product was collected. The black solid product was then washed with absolute alcohol for five to seven times followed by centrifugation. Finally, the solid product was dried in a desiccator for a few days for further characterization (sample 1). Other samples of bismuth nanomaterials were also prepared with different amounts of PVP and different solvents using the same methods under identical conditions (Table 1).

**Table 1:** Experimental conditions for the synthesis of bimuth nanomaterials

Sample number	The amount of PVP (g)	Solvent
**1**	0.155	EG
**2**	0	EG
**3**	0.310	EG
**4**	0.465	EG
**5**	0.155	DMF

### Characterizations

The bismuth nanostructures were characterized by powder X-ray diffraction (XRD), scanning electron microscopy (SEM), transmission electron microscopy (TEM), selected area electron diffraction (SAED), and energy-dispersive X-ray (EDX). The powder XRD pattern was recorded on Bruker AXS D8 Discover (Cu Kα radiation, λ = 1.5406 Å). The scanning rate is 1°/min in the 2θ range from 20 to 70°. The SEM images were taken on a Hitachi S4800 field emission scanning electron microscope operating at 3 or 5 kV (FESEM, Japan). TEM images and SAED pictures were recorded on a Philips Technai G2 20 electron microscope (Manufacturer: FEI Company), using an accelerating voltage of 200 kV. The EDX analysis was performed on an Oxford Instruments Inca with a scanning range from 0 to 20 keV. The samples for TEM observations were prepared by dispersing some of the solid products into absolute alcohol and then sonicated for several minutes. A few drops of the suspension were deposited on the copper grid, which was then put into the desiccator. The thermogravimetric and differential analysis was done by DIAMOND TG/DTA instrument (PerkinElmer Instruments, Waltham, CT, USA) with a heating rate of 10°C/min.

## Results and discussion

**Figure 1:**
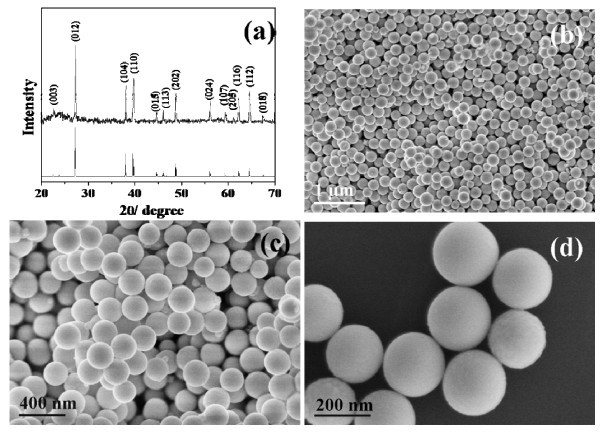
**XRD pattern (a) and SEM images of the as-synthesized product in EG at lower (b, c) and higher (d) magnifications (sample 1)**.

Figure 1a shows the typical powder XRD pattern of the as-synthesized product (sample 1). All the diffraction peaks could be readily indexed to be rhombohedral phase of bismuth, which were consistent with the literature values (JCPDS No. 05-0519). No impurity was detected in this pattern, indicating the formation of pure bismuth products under current synthetic condition. The sharp and strong diffraction peaks also confirm the well crystallization of the products.

The morphologies and structures of the as-prepared bismuth nanospheres were investigated by SEM and TEM images. Figure 1b, c, d shows the representative SEM images of the as-prepared bismuth nanospheres with different magnifications. The low-magnification images (Figure 1b, c) indicate the large production of uniform bismuth nanospheres. No other bismuth morphologies could be found in the whole sample. The diameters of these nanospheres varied from 160 to 210 nm and most of them were about 180 nm. The high-magnification image shown in Figure 1d clearly reveals the detailed morphologies of the products, indicating the formation of uniform, regular nanospheres with smooth surfaces.

**Figure 2:**
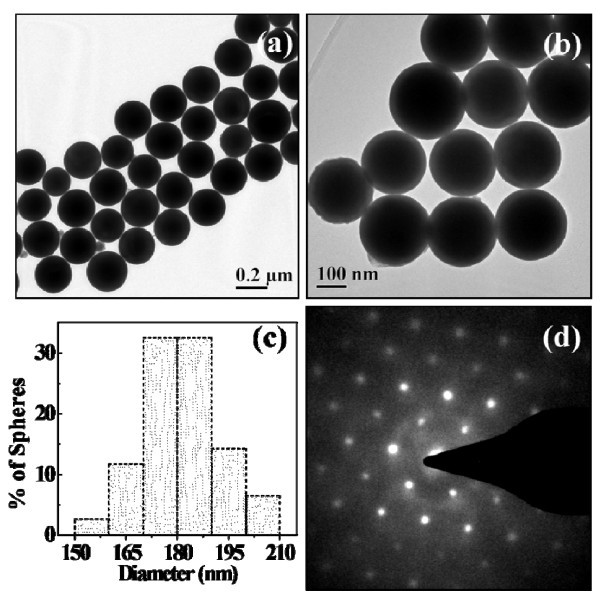
**Typical (a) low- and (b) high-magnification TEM images of as-prepared bismuth nanospheres (sample 1)**. **(c) **The histogram of size distribution of the bismuth nanospheres prepared from "a". **(d) **The SAED pattern of a single bismuth nanosphere.

The low- (Figure 2a) and high-magnification (Figure 2b) TEM images further demonstrated the morphologies of these bismuth nanospheres clearly. It also reveals a well-defined spheric structure and smooth surface. The statistical analysis showed that bismuth nanospheres had a narrow size distribution and the average diameter was about 180 nm with a standard deviation of 10 nm (Figure 2c). SAED pattern taken from an individual bismuth particle showed clear diffraction spots, indicating a good single-crystal structure (Figure 2d). The elemental composition of the nanospheres was also analyzed by EDX, and characteristic bismuth absorption peaks were observed (Figure S1 in Additional File 1). The copper and carbon peaks are due to the carbon-coated copper grid.

**Figure 3:**
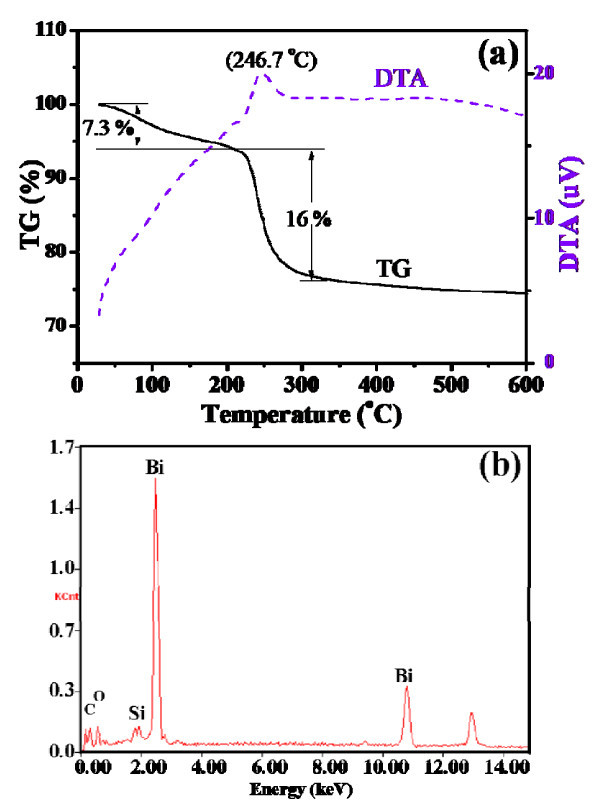
**Thermogravimetric and differential thermal analysis (TG/DTA) and EDX Analysis (a) TG/DTA curves of thermal decomposition of the white precipitation at a heating rate of 10°C/min in the N_2 _atmosphere; (b) EDX pattern of the white precipitation**.

To investigate the composition of the white precipitate obtained in the synthetic procedure, thermogravimetric and differential thermal analysis (TG/DTA) and EDX measurements were carried out. Figure 3a shows the results of the thermal analysis of the white precipitate at a heating rate of 10°C/min in the nitrogen atmosphere. It indicates a two-step weight loss in the temperature range from 25 to 200 and 200 to 300°C. The first weight loss in the TG trace could be attributed to the desorption of physically adsorbed water, and the second one could be recognized in according with the exothermal reaction in DTA trace (Figure 3a). The exothermal reaction thermal was found to arise from the decomposition of the white precipitate. The white precipitate was further investigated by EDX spectrum. As shown in Figure 3b, the white precipitate was composed of Bi, O, and C and no other additional elements were detected. The Si signal is attributed to the silicon wafers. When the white precipitate was calcined at 350°C for 90 min, the XRD pattern of the calcined product was very close to the standard data (Bi_2_O_3_, JCPDS No. 71-2274) (Figure S2 in Additional File 2). Hence, it was proposed that the possible component of the white precipitate was bismuth complexes (Bi^III^-mannitol), which were formed in ammonia solution.

In this study, it was found that there was no black product precipitated from the solution in the absence of PVP under identical experiment condition. The XRD pattern of the obtained white product could not be assigned in the XRD standard data base (Figure S3 in Additional File 3). It is obvious that PVP plays a key role in the formation of bismuth nanospheres. It was proposed that PVP reduced Bi^3+ ^ions to give metallic bismuth. The reduction process could be probably understood considering two main reactions: (1) direct abstraction of hydrogen atoms from the polymer by the Bi^3+ ^ions; (2) reduction of the Bi^3+ ^ions by the organic radicals formed in (1) or during the bismuth-accelerated degradation of PVP, which is similar to the reported literatures [40,42]. It was reported that the capping organic molecules in the reaction system could determine the subsequent morphology of the product because of their special chemical properties and self-assembling functions [43-45]. Recently, it was also found that EG molecules could adsorb onto the surface of bismuth crystals through O-Bi bonding which could significantly decrease their growth rates and lead to highly anisotropic growth [33,46]. Thus, when the molar ratio of repeating units of PVP to Bi^3+ ^is about 3, that is, there is no excess PVP in the solution, these uniform bismuth nanospheres can be obtained under the only effect of EG.(1)(2)

**Figure 4:**
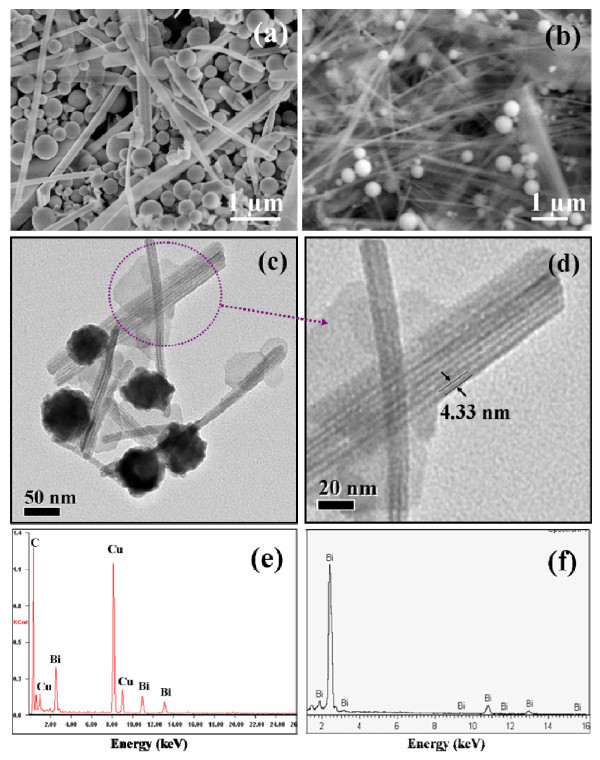
**SEM (a) image of bismuth nanomaterials were obtained when the amount of PVP was 0.310 g (sample 3)**. SEM image **(b) **and EDX pattern **(e) **of bismuth nanomaterials were obtained when the amount of PVP was 0.465 g (sample 4). Typical low- **(c)**, high-magnification **(d) **TEM images, and EDX spectrum (**f**) of the bismuth nanostructures using DMF instead of EG as solvent (sample 5).

When the amount of PVP was increased to 0.310 g (the molar ratio of repeating units of PVP to Bi^3+ ^is about 6), bismuth nanospheres and nanowires were formed, as shown in Figure 4a. The diameters of obtained bismuth nanospheres were in the range from 250 to 500 nm, and the length of the nanowires varies from 0.5 to more than 6 μm. When the molar ratio of repeating units of PVP to Bi^3+ ^was further increased to about 9 (the amount of PVP is 0.465 g), a mass of bismuth nanowires were obtained and few bismuth nanospheres remained, as shown in Figure 4b. The length of bismuth nanowires is up to 10 μm and the diameters of nanospheres are in range from 200 to 500 nm. The elemental composition of these nanowires and few nanospheres was also analyzed by EDX, and characteristic bismuth absorption peaks were observed (Figure 4e). Thus, PVP is considered to act both as reducing agent and capping agent in this synthesis. It was believed that the formation of bismuth nanowires was based on the Ostwald ripening process [47,48]. At first, bismuth nanoparticles were formed in the solution according to eqs 1 or 2 with PVP as reducing or nucleating agent. Then, the excess PVP molecules could cap onto the surface of bismuth particles [33], and bismuth nanowires were produced through this polyol process. In this process, bismuth particles can continuously grow into nanospheres, nanorods, or nanowires. The co-effect of PVP and EG resulted in a wide size distribution of the diameter of bismuth nanospheres in this experiment and literatures [31-33].

Interestingly, when DMF was instead of EG, bismuth nanoparticles and nanotubes arrays were formed under identical experimental conditions (Figure 4c, d). The average diameter of bismuth nanoparticles is about 50 nm, and the diameters of the bismuth nanotubes are in a narrow range from about 3.2 to 5.2 nm, with an average diameter of 4.3 nm. EDX was used to further analyze the elemental compositions of these nanotubes lying on the hole of the copper grid. It reveals the elemental Bi signals and confirmed the purity of this bismuth nanotube (Figure 4f). The copper and carbon peaks are due to the carbon-coated copper grid. Here, DMF plays an important role in the fabrication of bismuth nanotubes. In this synthesis, the starting materials can dissolve in DMF and DMF can partially decompose into dimethylamine, which is a good coordination ligand with N-chelation. The diffusion of Bi^3+ ^ions in DMF-dimethylamine will be faster due to its lower viscosity, which is beneficial for the formation of nanotube products [49]. The formation mechanism is still limited and further study in progress.

## Conclusions

In summary, uniform bismuth nanospheres were successfully synthesized via a facile solvothermal process by reduction of EG solution of bismuth salt, using PVP both as a reducing agent and a capping agent. The shapes and sizes of the bismuth nanostructures varied with the changing of the PVP/bismuth salt molar ratio. Uniform bismuth nanospheres with an average diameter of 180 nm could be routinely synthesized through this solvothermal approach by controlling the PVP/bismuth salt ratio. The solvent EG also played an important role in the formation of uniform bismuth nanospheres. Bismuth nanoparticles and nanotubes were formed when DMF was used as a solvent instead of EG. The possible formation mechanism of the bismuth nanotubes was also discussed and further study was in progress.

## Abbreviations

DMF: *N*,*N*-dimethylformamide; EDX: energy-dispersive X-ray; EG: ethylene glycol; PVP: poly(*N*-vinyl-2-pyrrolidone); SAED: selected area electron diffraction; SEM: scanning electron microscopy; TEM: transmission electron microscopy; XRD: X-ray diffraction.

## Competing interests

The authors declare that they have no competing interests.

## Authors' contributions

JW participated in the acquisition of data and drafted the manuscript. FQ carried out the SEM characterization and participated the analysis and interpretation of data. ZL and HJY helped to draft and revise the manuscript. RC made contributions to conception and design of the study, revised the manuscript critically for important intellectual content, and has given final approval of the version to be published.

## Supplementary Material

Additional file 1 **Figure S1**. EDX spectrum of bismuth nanospheres prepared in EG. Click here for file

Additional file 2 **Figure S2**. XRD pattern of the white precipitation was calcined at 350°C for 90 min. Click here for file

Additional file 3 **Figure S3**. XRD pattern of the white product obtained in EG in the absence of PVP. Click here for file
