# Supplementary figures and images for: Solvothermal synthesis of uniform bismuth nanospheres using poly(N-vinyl-2-pyrrolidone) as a reducing agent
Source: Nanoscale Res Lett. 2011 Jan 12;6(1):66. doi: 10.1186/1556-276X-6-66 (PMC3212213; doi:10.1186/1556-276X-6-66)

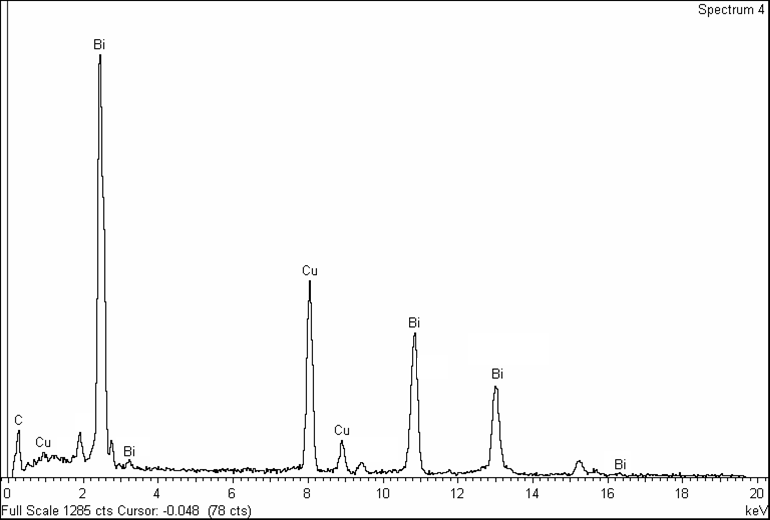


Fig. S1 EDX spectrum of bismuth nanospheres prepared in EG.

Supplement: Additional file 1 — Figure S1. EDX spectrum of bismuth nanospheres prepared in EG. [file 1556-276X-6-66-S1.DOCX]
