# Supplementary material for: Solvothermal synthesis of uniform bismuth nanospheres using poly(N-vinyl-2-pyrrolidone) as a reducing agent
Source: Nanoscale Res Lett. 2011 Jan 12;6(1):66. doi: 10.1186/1556-276X-6-66 (PMC3212213; doi:10.1186/1556-276X-6-66)
Supplement: Additional file 2 — Figure S2. XRD pattern of the white precipitation was calcined at 350°C for 90 min. [file 1556-276X-6-66-S2.DOCX]

Fig. S2. XRD pattern of the white precipitation was calcined at 350 °C for 90 min.
