# Supplementary material for: Solvothermal synthesis of uniform bismuth nanospheres using poly(N-vinyl-2-pyrrolidone) as a reducing agent
Source: Nanoscale Res Lett. 2011 Jan 12;6(1):66. doi: 10.1186/1556-276X-6-66 (PMC3212213; doi:10.1186/1556-276X-6-66)
Supplement: Additional file 3 — Figure S3. XRD pattern of the white product obtained in EG in the absence of PVP. [file 1556-276X-6-66-S3.DOCX]

Fig. S3. XRD pattern of the white product obtained in EG in the absence of PVP.
